# Feasible Route for a Large Area Few-Layer MoS_2_ with Magnetron Sputtering

**DOI:** 10.3390/nano8080590

**Published:** 2018-08-03

**Authors:** Wei Zhong, Sunbin Deng, Kai Wang, Guijun Li, Guoyuan Li, Rongsheng Chen, Hoi-Sing Kwok

**Affiliations:** 1 School of Electronic and Information Engineering, South China University of Technology, Guangzhou 510640, China; zwnice@163.com (W.Z.); phgyli@scut.edu.cn (G.L.); 2 State Key Laboratory on Advanced Displays and Optoelectronics Technologies, Department of Electronic and Computer Engineering, The Hong Kong University of Science and Technology, Hong Kong, China; sdengaa@connect.ust.hk (S.D.); kaiwjnu@163.com (K.W.); gliad@connect.ust.hk (G.L.); eekwok@ust.hk (H.-S.K.)

**Keywords:** few-layer MoS_2_, magnetron sputtering, magnetron sputtering power, raman spectroscopy, disorder

## Abstract

In this article, we report continuous and large-area molybdenum disulfide (MoS_2_) growth on a SiO_2_/Si substrate by radio frequency magnetron sputtering (RFMS) combined with sulfurization. The MoS_2_ film was synthesized using a two-step method. In the first step, a thin MoS_2_ film was deposited by radio frequency (RF) magnetron sputtering at 400 °C with different sputtering powers. Following, the as-sputtered MoS_2_ film was further subjected to the sulfurization process at 600 °C for 60 min. Sputtering combined with sulfurization is a viable route for large-area few-layer MoS_2_ by controlling the radio-frequency magnetron sputtering power. A relatively simple growth strategy is demonstrated here that simultaneously enhances thin film quality physically and chemically. Few-layers of MoS_2_ are established using Raman spectroscopy, X-ray diffractometer, high-resolution field emission transmission electron microscope, and X-ray photoelectron spectroscopy measurements. Spectroscopic and microscopic results reveal that these MoS_2_ layers are of low disorder and well crystallized. Moreover, high quality few-layered MoS_2_ on a large-area can be achieved by controlling the radio-frequency magnetron sputtering power.

## 1. Introduction

The emergence of monolayer graphene [1,2] and transition metal dichalcogenides (TMDs) [3,4] has inspired a series of high-profile discoveries in the electronic and optoelectronic fields [5,6,7], and has initiated potentially new areas [8,9]. Thus, two-dimensional (2D) materials have recently been intensively studied. In the context of 2D TMDs, molybdenum disulfide (MoS_2_) is one of the attractive embodiments due to its stable form in few- and single-layers [10,11] as well as its desirable electrical and optical properties [12]. Few- and single-layer MoS_2_ is firstly obtained via top-down mechanical stripping techniques [13], which are commonly used for graphene exfoliation. Although this method of exfoliation and dip coating [14,15,16,17] has its own the advantages to achieve high-quality 2D materials, none of these are proper solutions for radical large-scale commercial manufacturing, where the mass-producible growth of large-area, continuous, and high-quality 2D MoS_2_ thin films on dielectrics is a pre-requisite. From this point of view, the bottom-up strategies for thin film growth, including chemical vapor deposition (CVD) and physical vapor deposition (PVD), are better choices when compared with the top-down methods mentioned above. CVD methods have already been successfully demonstrated [18,19,20], but the control of thin film thickness, purity and uniformity on a large scale needs to be further enhanced [21]. On the other hand, PVD methods, especially magnetron sputtering, have been broadly employed in large-scale commercial manufacturing at low cost and with easy control. However, the exploration of 2D MoS_2_ thin film growth using magnetron sputtering technology is quite insufficient [21]. In recent years, there have been few attempts at sputtering techniques for the growth of MOS_2_ thin films. Muratore et al. [22], Kaindl et al. [23], and Samassekou et al. [24] reported the synthesis of continuous few-layer MoS_2_ by sputtering method using a MoS_2_ target, and the sputtered MoS_2_ films was annealed in an argon atmosphere. Tao et al. [21] and Santoni et al. [25] reported MoS_2_ film using Mo target sputtered in vaporized sulfur ambient. However, the reported films either are relatively thick or have poor crystal quality and optical properties [21,22,23,24,25]. The main obstacles for large-area sputtered high quality MoS_2_ films are possibly located in the difficulty of disorder control during thin film deposition, and the lack of metrics to evaluate the deposited thin films. 

Studies over the past decade have shown that Raman spectroscopy has historically played an important role in the structural characterization of graphitic materials [26,27,28] and has also become a powerful tool to understand the effects of process parameters on the production of high quality graphene by monitoring changes in disordered peaks [28,29,30,31,32]. Recently, Raman spectroscopy has also been used to study the effects of disorder on the MoS_2_ [33]. On the other hand, since many scholars have previously investigated the crystallinity of MoS_2_ thin film by high-temperature vulcanization or deposition on different substrates [21,34,35], few studies have investigated the effect of sputtering power on the MoS_2_ thin film. Therefore, in this work, we use a Raman spectroscopy approach to describe the quality and to study the effect of RF power on the deposition of the large-scale few-layer MoS_2_ films. We also investigated the crystalline structure of the films through an X-ray diffractometer (XRD) and a high-resolution field emission transmission electron microscope (HRTEM). The binding energies of Mo in the MoS_2_ film grown by radio frequency magnetron sputtering (RFMS) were further analyzed by X-ray photoelectron spectroscopy (XPS). For precise detection, the surface 5-nm-thick thin films were etched using Ar ions before XPS characterization.

## 2. Materials and Methods

**Figure 1:**
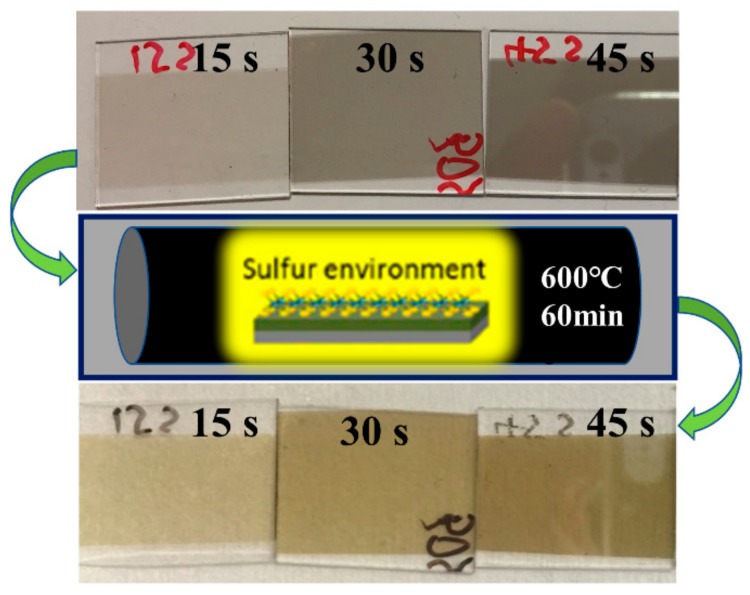
This post-deposition annealing treatment was performed to further enhance crystalline quality in as-sputtered MoS_2_ on glass substrates under sulfur environment.

The large-scale few layer MoS_2_ thin films were deposited using the RFMS technique. Firstly, both silicon substrates coated with thermally grown SiO_2_ and glass substrates (Eagle 2000, Corning) were ultrasonically cleaned in acetone and then isopropanol (IPA). After being rinsed in deionized (DI) water and dried, the substrates were loaded into the chamber of an RFMS system (AJA International Inc., Scituate, MA, USA) and heated to 400 °C. The distance between the substrate and the MoS_2_ target (99.99%, 2 Inc., Plasmaterials Inc., Livermore, CA, USA) was 10 cm. In order to suppress the influence of oxygen and moisture on the deposited thin films, the base pressure of the chamber should be pumped down as low as possible. In this work, the value was 2.67 × 10^−5^ Pa. During deposition, Ar gas (99.999%) was allowed to flow into the chamber with a stable flow rate of 20 sccm, and the working pressure of the chamber was maintained at 3 × 10^−3^ Torr. The RF sputtering power applied to the MoS_2_ target was varied from 10 W to 150 W in order to investigate the relationship between the large-scale few layer MoS_2_ growth and RF power. The deposition time was dependent on the required thickness of the thin films and the thickness of the film is maintained at 15 nm, the thickness of the thin films is confirmed by HRTEM (High-Resolution Transmission Electron Microscopy). When the RFMS process was completed, the heater and the Ar gas pipeline were switched off, and the substrates were cooled down to room temperature naturally. After loading out of the chamber, the SiO_2_/Si substrates and glass substrates with as-deposited MoS_2_ thin film were immediately subjected to post-annealing in a sulfurization atmosphere for 60 min at 600 °C to enhance thin film quality physically and chemically (Figure 1).

In order to identify the layered structure and obtain the disorder information, the deposited MoS_2_ thin films were analyzed using Raman spectroscopy (Renishaw invia RE04, 514 nm Ar laser with a 1 μm spot size, Renishaw plc, Gloucestershire, United Kingdom). For the crystalline phase characterizations of the thin films, an X-ray diffractometer (XRD, Empyrean, PANalytical, Almelo, The Netherlands) with Cu Kα radiation was used. It was operated in thin-film mode, and the angle between the X-ray and the thin film surface was fixed at 0.5 degrees. Furthermore, a high-resolution transmission electron microscopy (HRTEM, JEM-2010HR, JEOL, Tokyo, Japan) was applied to identify the number of layers and atomic structure of the MoS_2_ film. To analyze the material composition of the deposited thin films as well as the chemical environment of the atoms in the thin films, X-ray photoelectron spectroscopy (XPS) measurement was conducted on the Physical Electronics 5600 multi-technique system (Physical Electronics Inc., Chanhassen, MN, USA).

## 3. Results and Discussion

Figure 1 shows an image of MoS_2_ thin layer grown on glass substrates with different deposition time (15 s, 30 s, and 45 s). The deposited MoS_2_ layer was light gray, and after annealing under a sulfur atmosphere, the MoS_2_ layer was pale yellow and was found to have specular reflection of ambient light. This result is consistent with previous reports [21]. The size of the synthesized films is limited by the dimensions of our sample heating holder.

**Figure 2:**
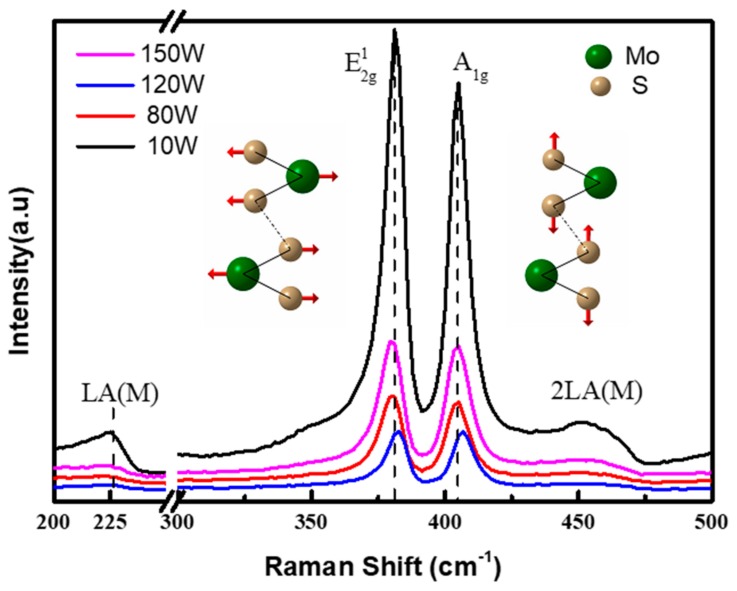
Raman spectra of the MoS_2_ thin films deposited on SiO_2_/Si substrates under different radio frequency (RF) powers. The insets illustrate the oscillating mode of the $E_{2g}^{1}$ and A_1g_ peak.

Figure 2 shows the Raman spectra of the MoS_2_ thin films on SiO_2_/Si substrates deposited under different RF powers of 10 W, 80 W, 120 W and 150 W, respectively. From Figure 2, it can be seen that all of the thin films exhibit two specific Raman characteristic peaks of MoS_2_, namely the in-plane ($E_{2g}^{1}$) peak at ~381 cm^-1^ and the out-of-plane (A_1g_) peak at ~405 cm^−1^. Their peak positions herein are quite consistent with the results in other reports [24,36,37] , which the $E_{2g}^{1}$ and A_1g_ mode peaks appear at ~ 381.5 cm^−1^ and ~404.8 cm^−1^. Besides this, a relatively small LA(M) peak (a defect peak generated by LA phonons at the M point in the Brillouin zone) is also observed at ~227 cm^−1^. Theoretically, the $E_{2g}^{1}$ mode corresponds to the S and Mo atoms oscillating in antiphase parallel to the crystal plane, while the A_1g_ mode corresponds to the S atoms oscillating in antiphase out-of-plane, as shown in the insets of Figure 2 [29,38]. In addition to the absolute positions of these two peaks, the frequency difference (Δk) between the A_1g_ peak and the $E_{2g}^{1}$ peak is a good indicator of the layer number in MoS_2_ thin films. In this work, Δk is around 24 cm^-1^, which is larger than that in monolayer MoS_2_ thin films (~18–19 cm^−1^) [19,33,39], but smaller than the typical value in bulk MoS_2_ (~26 cm^−1^) [37,38]. This indicates the existence of few-layer MoS_2_ [36]. Table 1 lists the peak positions corresponding to the $E_{2g}^{1}$ and A_1g_ mode as well as the Δk values for all samples under various RF sputtering powers in this work. With the continuous increase of RF power from 10 W to 150 W, the Δk values remain in the vicinity of 24 cm^−1^. This means that all of the thin films under different RF powers are MoS_2_ with a few layers [36,37]. Another indicator of film quality is the full width at half maxima (FWHM) of the observed vibration modes. FMHM values for the sputtered FL-MoS_2_ film with different RF powers is compared in Table 1. In general, higher FMHM values mean more disorder [24,25]. From the Table 1, it can be seen that the MoS_2_ films with RF powers of 120 W has the lowest FMHM values, meaning it has the least disorder. It also can be seen that the A_1g_ peak and the $E_{2g}^{1}$ peak all have a blue shift as the RF powers increased from 10 W to 120 W and a red shift as the RF powers increased from 120 W to 150 W. The red shift is attributed to the high RF power, resulting in an increase in the residual-stress of the film [40].While the blue shift is attributed to O_2_-doping of MoS_2_, which will be shown on the XPS result [41].

**Figure 3:**
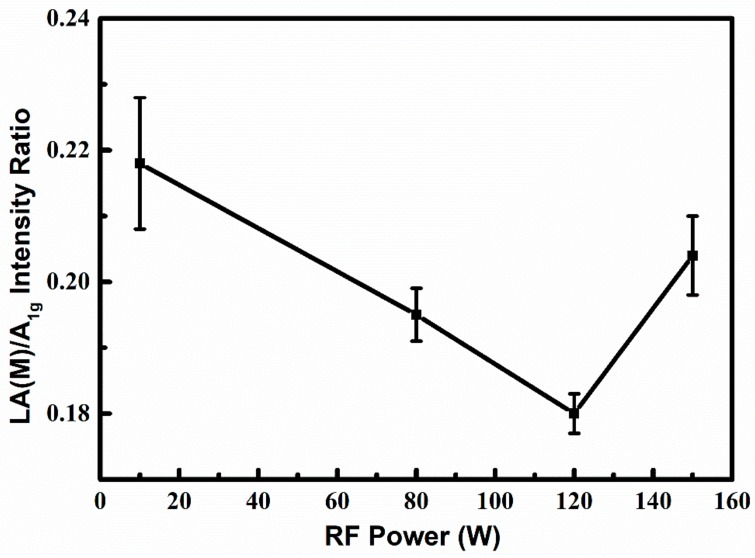
LA(M) to A_1g_ peak intensity ratio of the MoS_2_ films with different RF powers deposited on SiO_2_/Si substrates.

According to Raman fundamental selection rules, only phonons with wave vector q ≌ 0 are Raman active around the center of the Brillouin zone. However, this rule will be broken by defects, which cause the appearance of peaks away from the zone center [33,42]. Monitoring the evolution of disorder-related sub-peaks in the Raman spectrum enables us to understand the effects of process parameters and has allowed great strides to be made in the CVD preparation of high quality graphene [29,43,44]. Similarly, in Figure 2, apart from two major peaks with regard to the basic $E_{2g}^{1}$ and A_1g_ vibration modes, several sub-peaks related to the defects can also be observed. Among them, the sub-peak at 227 cm^−1^ is the most intense, which is attributed to the longitudinal phonon branch at the point M (LA (M)) of the Brillouin region [33,45]. Therefore, this sub-peak is able to form a very clear marker for disorder in the system, especially for the few- and single-layer MoS_2_. The intensity ratio of the LA(M) sub-peak to A_1g_ peak is plotted as a function of RF power in Figure 3. It can be clearly observed that the intensity ratio reaches a minimum when the RF power climbs to 120 W, indicating the improvement of thin film quality [46]. In general, higher RF power could assist with the formation of crystalline films with lower disorder (namely, lower LA(M)/ A_1g_ intensity ratio), but excessively high RF power is not welcomed. For instance, the LA(M)/A_1g_ intensity ratio rises when the RF power increases from 120 W to 150 W. It reveals the increase of disorder in the deposited MoS_2_ thin films. A possible explanation for this phenomenon could be the increased defect generation caused by ion bombardment under over-high RF power.

**Figure 4:**
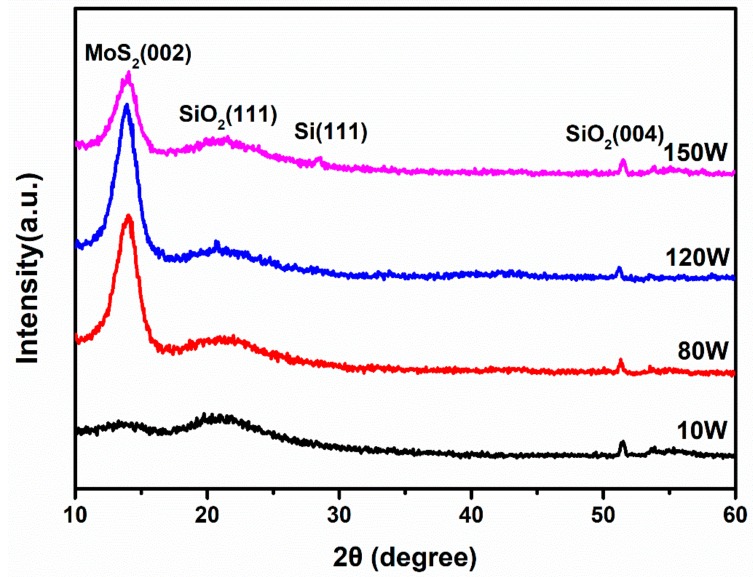
X-ray diffraction patterns of the MoS_2_ thin films on SiO_2_/Si substrates under different RF sputtering powers.

The crystalline information of the samples was characterized using XRD. The X-ray diffraction patterns of MoS_2_ thin films on SiO_2_/Si substrates under different RF powers are shown in Figure 4. The MoS_2_ thin films deposited under different RF powers all exhibit three obvious diffraction peaks, which are located at 14.0°, 21.6°, and 51.5° respectively. The two weak diffraction peaks at 21.6° and 51.5° correspond to the SiO_2_ (JCPDS: 27-0605) (111) and (400) planes, respectively. The strong diffraction peak at 14.0° is an indicator of the MoS_2_ (JCPDS: 37-1492) (002) plane. For the MoS_2_ thin films deposited under an RF power of 10 W, the broad and weak diffraction peak at 14.0° indicates the amorphous structure of the film. However, when the RF power increases to 80 W, 120 W and then 150 W, all of the MoS_2_ thin films exhibit a strong and narrow diffraction peak at 14.0°, which indicate the well crystallization of MoS_2_ thin films. Since the (002) plane is parallel to the surface of the substrates, the deposited MoS_2_ thin films using the RFMS technique under an RF power of 80 W, 120 W and 150 W could grow along the c-axis. Meanwhile, the exclusive diffraction peak also suggests the thin films are highly oriented. These properties are quite helpful for the formation of stacked microstructures in MoS_2_ thin films. Besides this, it should be noted that the intensity of the (002) peak for MoS_2_ thin films under an RF power of 150 W is lower compared to the other two samples. This is possibly related to the degradation of crystalline quality under such high RF powers [47]. At the same time, we also noticed that under the RF power of 150 W, the sample showed a weak diffraction peak at 28.4°, which corresponds to the Si (JCPDS: 27-1402) (100) plane.

**Figure 5:**
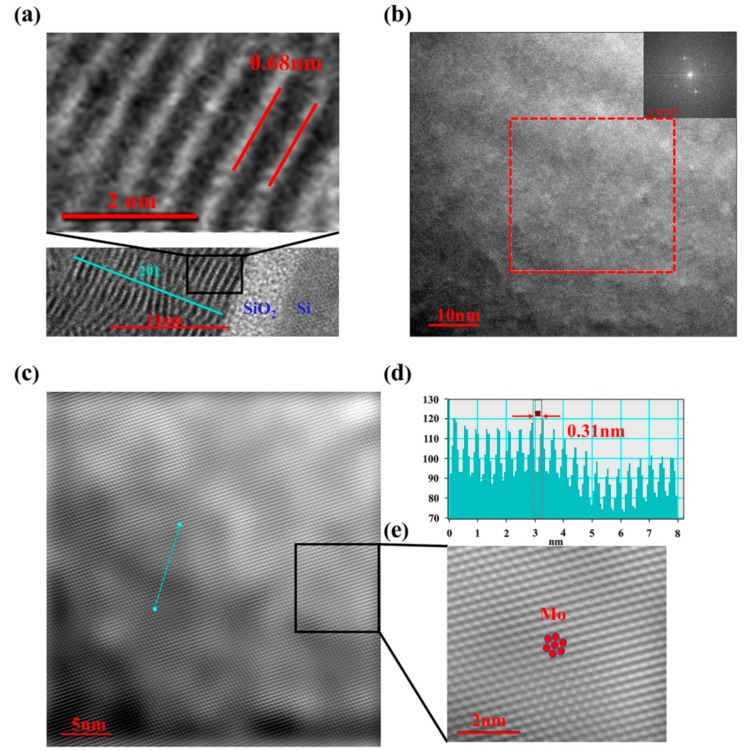
(**a**) Cross-sectional high-resolution field emission transmission electron microscope (HRTEM) image of samples deposited under an RF power of 120 W. (**b**) High resolution TEM image of the MoS_2_ film deposited by 120 W RF power transferred onto a lacey carbon grid. The inset shows the fast Fourier transformation (FFT) image corresponding to the TEM image selected area of a portion of (**b**), showing the hexagonal symmetry of the MoS_2_ structure. (**c**) Inverse FFT images corresponding to the TEM image selected area of a portion of (**b**). (**d**) Atomic spacing along the selected direction of the basal plane. (**e**) Zoom-in image of the area highlighted in (**c**). The hexagonal structure formed by Mo atoms is indicated.

To further elucidate the crystalline structure, the MoS_2_ thin film deposited by 120 W RF power was transferred onto a lacey copper grid for HRTEM characterizations. Figure 5a presents its cross-sectional HRTEM image, it can be seen that there are about 20 layers of the MoS_2_ thin films on the SiO_2_/Si substrate, and the interlayer spacing (0.68 nm) of the MoS_2_ thin films is consistent with the previous results [21]. Moreover, it is also verified that the deposited MoS_2_ thin films are stacked a few layers in parallel, which is in agreement with the results extracted from the Raman spectra above. As far as the typical high resolution TEM image in Figure 5b is concerned, the first-order diffraction spots of the FFT image (inset of Figure 5b) on a selected area are shown according to a regular hexagonal symmetry, thus indicating the presence of the MoS_2_ thin film made of single crystal domains (without Moireé patterns). Moreover, HRTEM images of the selected area in Figure 5b after FFT filtering are shown in Figure 5c. The IFFT-filtered image (Figure 5c) shows a regular honeycomb pattern due to the atomic arrangements of the Mo and S atoms. In Figure 5d, the calculated profile along the selected direction in Figure 5c is drawn, which reveals the (004) plane of MoS_2_ with a lattice spacing of 0.31 nm that is observed in Figure 5c. Figure 5e is the magnified image of the square-surrounded region in Figure 5c. The periodic atom arrangement for Mo confirms that the MoS_2_ thin films deposited under an RF power of 120 W own a crystalline structure. This is also consistent with the XRD results in Figure 4.

**Figure 6:**
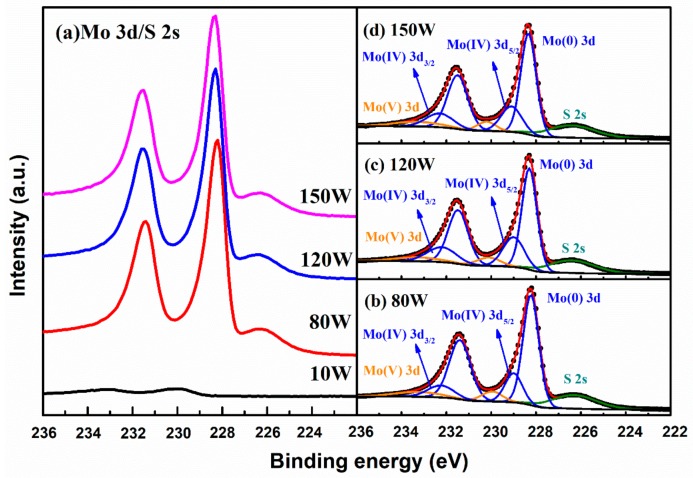
High-resolution X-ray photoelectron spectroscopy (XPS) spectra of (**a**) Mo 3d/S 2s, and (**b**), (**c**), and (**d**) Mo3d core-level spectra for an RF power of 80 W, 120 W, and 150 W, respectively. The background is shown with a black line at the bottom. The black dots represent the raw data. The red line is the total least-squares fit. The orange lines indicate the Mo(V) 3d components. The blue lines are the Mo3d components linked to Mo (0) and Mo (IV), the Mo (0) are the lower BE doublet. The olive lines are the S2s components. (See text for more explanation).

**Figure 7:**
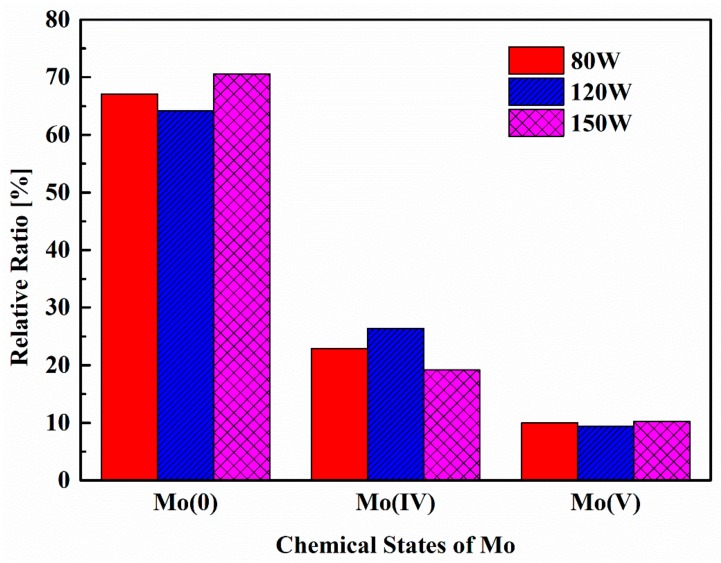
Relative ratio of Mo species with various chemical states at different RF powers.

XPS was used to analyze the chemical environment of Mo in the MoS_2_ thin films, and to detect any impurity (particularly oxygen) involvement during preparation. The high-resolution XPS spectra of the Mo 3d and S 2s region are shown in Figure 6. Since the peaks of Mo 3d and S 2s are too close to distinguish, in order to obtain the chemical environment information of the Mo species, both the S 2s and Mo 3d spectra are taken into account. Moreover, since the main Mo doublet peak signals of the thin films under an RF power of 10 W are so weak that they reach noise level, the analysis in terms of such samples is not reliable. Hence, only the data with an RF power of 80 W, 120 W and 150 W have been fitted by a 20% Lorentzian–Gaussian ration fit and the according results together with the Shirley background are presented in Figure 6b–d. According to [35], the two peaks at 229.1 eV and 232.2 eV for the MoS_2_ thin film are attributed to the doublet Mo 3d_5/2_ and Mo 3d_3/2_ orbitals, respectively. Meanwhile, the fitting shows that there is a second characteristic at the lower binding energy. For species originating from lower binding energy (BE) Mo3d peaks, the lower BE Mo species is Mo still associated with the Mo-S lattice, or it reflects a single amorphous MoS_x_ phase, where Mo has a different number of nearest neighbor S atoms [48,49]. According to [25], the lower BE Mo species can be assigned to zero-valent Mo (Mo(0)) occurring in small aggregates dispersed in a MoS_2_ matrix. From the principle of sputter coating, we can know that the presence of Mo(0) cannot be avoided, and the purpose of annealing under a sulfur atmosphere is to convert Mo(0) to Mo(IV), which can reduce disorder and defect formation. However, when the film is etched by an ion beam, there is also a chemical shift of its binding energy toward smaller values [25,35]. Therefore, compared to XPS characterization, the advantages of strong non-destructive characterization of Raman spectra are even more pronounced. For accurate detection, 5-nano-thin-thick-surface films should be etched with Ar ions prior to XPS characterization. However, this will lead to sample destruction and will have a certain impact on the test results. Although this changed the chemical state of the Mo atom, all the samples were processed in the same way, and the overall trend of its variation with sputtering power did not change. The relative ratio of Mo species calculated from the deconvolved Mo 3d spectrum results is shown in Figure 7. The Mo(IV) fraction is defined as the area under the Mo(IV) peak divided by the total Mo peak area. It can clearly be seen that the Mo (IV) fraction reaches a maximum when the RF power is increased to 120 W, indicating the improvement of thin film quality. In addition, smaller peaks at 230.2 eV and 233.3 eV are assigned to Mo^5+^ [50], indicating the presence of chemisorbed oxygen at sulfur vacancies [51,52] or sub-stoichiometric oxides MoO_x_ [53]. The origin of MoO_x_ is probably due to the sulfurization process. We believe that MoO_x_ has probably formed during the deposition and/or during the sulfurization process at 600 °C by reaction with the oxygen diffused from the SiO_2_/Si substrate. As shown in Figure 7, the Mo (V) fraction is around 0.1, 0.094 and 0.102 for an RF power of 80 W, 120 W, and 150 W, respectively. For different RF powers, the Mo(V) fraction is basically the same, indicating that the power change has no effect on the Mo(V) fraction.

## 4. Conclusions

Few-layer MoS_2_ thin film deposition on large-area thermally oxidized silicon substrates was demonstrated using the RFMS technique. Raman analysis verified the achievement of few-layer MoS_2_ thin films. Meanwhile, it was proposed that the disorder inside the thin films could be monitored using Raman spectra, and controlled by adjusting the RF sputtering power. Furthermore, the XRD spectra and cross-sectional TEM images confirmed the high quality of few-layer MoS_2_ thin films, implying that RFMS was suitable for layered MoS_2_ growth. Additionally, the XPS characterizations on RFMS grown few-layer MoS_2_ thin films revealed the RF power has a great effect on the binding energies of Mo atoms. Our work illustrates that sputtering combined with sulfurization is a viable route for the high quality of large-area few-layer MoS_2_ by controlling the radio-frequency magnetron sputtering power.

## Figures and Tables

**Table 1:** The $E_{2g}^{1}$- and A_1g_-related Raman peak information of MoS_2_ thin films deposited using radio frequency magnetron sputtering (RFMS) under various RF powers.

RF Power (W)	A_1g_ (cm^−1^)	$E_{2g}^{1}$ (cm^−1^)	Δk (A_1g_-$E_{2g}^{1}$) (cm^−1^)	Full Width at Half-Maximum (cm^−1^)		LA(M) to A_1g_ Peak Intensity Ration
				A_1g_	$E_{2g}^{1}$	
10	405.2 ± 0.1	381.2 ± 0.1	24.0 ± 0.02	10.64 ± 0.46	10.87 ± 0.56	0.219 ± 0.010
80	405.4 ± 0.4	381.4 ± 0.4	24.0 ± 0.01	10.17 ± 0.34	10.31 ± 0.21	0.195 ± 0.004
120	407.0 ± 0.1	383.0 ± 0.1	24.0 ± 0.02	9.55 ± 0.02	9.57 ± 0.20	0.180 ± 0.003
150	403.7 ± 0.4	379.7 ± 0.4	24.0 ± 0.02	10.49 ± 0.13	10.56 ± 0.02	0.204 ± 0.006
